# Assessing the effect of On-site supportive communication training (On-site SCT) on doctor burnout: a randomized controlled trial

**DOI:** 10.1186/s12909-025-06710-0

**Published:** 2025-01-23

**Authors:** K.K. Antonsen, J.D. Lyhne, A.T. Johnsen, S. Eßer-Naumann, L.Ø. Poulsen, L. Lund, S. Timm, L.H. Jensen

**Affiliations:** 1 Department of Oncology, Vejle Hospital, University Hospital of Southern Denmark, Vejle, Denmark; 2 Department of Regional Health Research, University of Southern Denmark, Odense, Denmark; 3 Department of Oncology and Palliative Care, Zealand University Hospital, Roskilde/Naestved, Denmark; 4 Department of Oncology, Aalborg University Hospital, Aalborg, Denmark; 5 Center for Shared Decision Making, Vejle Hospital, University Hospital of Southern Denmark, Vejle, Denmark

**Keywords:** Self-assessment, Communication skills training, Doctor-patient communication, Continuing professional education, Continuing professional development, Feasibility

## Abstract

**Background:**

Burnout is a critical factor that can influence the quality of care that doctors provide to their patients. Previous research suggests a link between inadequate communication skills training and burnout, and various approaches to enhance communication skills have been explored as a means to address this issue. However, evidence of the effect of these approaches is lacking. The aim of this study is to assess the effect of the novel On-site Supportive Communication Training (On-site SCT) in enhancing communication skills among oncologists and thereby addressing burnout.

**Methods:**

This randomized, controlled, multicenter study was conducted across three oncological departments in Denmark. Doctors were eligible if they worked in the outpatient clinic at least four days per month and provided informed consent. Doctors in the intervention group underwent a two-hour introduction followed by three full days of On-site SCT facilitated by in-house psychologists, while those in the control group continued standard practices. Pre- and post-intervention assessments on burnout (Copenhagen Burnout Inventory) were conducted, as were assessments of related constructs (job satisfaction and communication self-efficacy). Differences in pre- and post- assessments were analyzed using a paired t-test. Feasibility was assessed descriptively by comparing intervention days with planned schedule, and doctors’ satisfaction with the intervention was assessed systematically by questionnaire.

**Results:**

Of 101 screened doctors, 89 (88%) consented and were randomized. 65% were female, and the mean age was 46 (range 27 to 75). Due to nine exclusions, data from 39 doctors in the intervention group and 41 doctors in the control group were available for analysis. At baseline, doctors exhibited lower levels of burnout than reported in international literature. No statistically significant improvements in burnout (*p* > 0.05) were demonstrated post-intervention. Despite non-significant changes, the doctors reported an improvement in communication self-efficacy. The program showed high feasibility and received positive feedback from participating doctors.

**Conclusions:**

Our findings caution against assuming a causal relationship between short-term interventions and a complex phenomenon like burnout. On-site SCT demonstrated high feasibility, participation rate and acceptance. This underscores its potential value in clinical settings. Consequently, On-site SCT will be implemented at the Department of Oncology, Vejle University Hospital, to facilitate further refinement based on ongoing feedback and to explore long-term outcomes.

**Trial registration:**

December 2022– The Region of Southern Denmark (22/57137). April 2023– ClinicalTrials.gov (NCT05842083). April 2023– The Research Ethics Committee at the University of Southern Denmark (23/19397).

## Background

To ensure the highest quality of cancer care, treatment should address not only the unique characteristics of the cancer but also the individual needs and preferences of each patient [1, 2]. Achieving this understanding requires clear and open communication between healthcare providers and patients. Such communication is highly complex, involving various aspects, from delivering information about the cancer diagnosis, treatment options, and prognosis to fostering patient understanding and shared decision-making [2]. To achieve effective communication, the doctor must establish an interpersonal relationship with the patient that addresses emotional needs, manages uncertainties, and provides unwavering support throughout the entire cancer journey [3, 4]. Therefore, caring for patients with cancer may represent one of the most demanding tasks for a doctor in the field of medicine [5].

Burnout, first conceptualized in the 1970s by Freudenberger [6], is described as a psychological and physical syndrome resulting from prolonged exposure to excessive demands on energy, strength, and personal resources in a professional context. It manifests through symptoms such as emotional exhaustion, depersonalization, and a diminished sense of personal accomplishment [6]. Freudenberger emphasized the occupational nature of burnout, highlighting its prevalence among those in helping professions driven by strong ideals and a deep sense of commitment. Burnout has been formally defined in the International Classification of Diseases (ICD-11) as “resulting from chronic workplace stress that has not been successfully managed.” Additionally, the World Health Organization (WHO) specifies that burnout refers to “a phenomenon in the occupational context” [7], i.e., a job-specific syndrome.

The demanding nature of oncology exposes oncologists to profound existential questions, heightening their susceptibility to burnout syndrome [8, 9]. A 2017 meta-analysis revealed that 32% of oncology professionals experienced “high burnout” [10], a prevalence comparable to a Danish study on palliative physicians [11], where 23% reported symptoms of work-related burnout requiring intervention. However, only 4% of the palliative physicians reported symptoms of client-related burnout requiring response [11]. A recent scoping review of 17 studies among young European oncologists found burnout prevalence rates as high as 52–78%, emphasizing the persistent challenge of burnout within the oncology profession [12]. Due to its high prevalence, the implications of burnout in oncology are considerable. For the individual, long-term burnout might lead to personal consequences such as physical and/or mental health issues [8]. In a professional context, sustained burnout can result in decreased quality of care [8], decreased efficacy, errors, and decreased productivity among clinical oncologists [12]. At an organizational level, oncologists’ well-being is paramount for the success of practices, as burnout significantly affects care quality, safety, and patient satisfaction, while also impacting recruitment and retention [8]. Additionally, research suggests a strong association between burnout and job satisfaction [12, 13].

### Strategies for mitigating burnout

Numerous studies have explored the causes and management of burnout in clinical oncology. Although causation cannot be definitively established, research has suggested three main areas associated with burnout: demographic factors (e.g., age, living alone), individual factors (e.g., higher loan debt or having a psychosomatic disorder), and work factors (e.g., workload) [9, 14]. Since demographic factors can be difficult to address in a professional context, individual and work factors often become the primary focus when designing interventions addressing physician burnout. In 2016, West et al. conducted a systematic review and meta-analysis of interventions addressing burnout [15]. These interventions typically fall into two categories: individual focused and/or organizational focused [9, 12, 15, 16]. Individual focused interventions include small group curricula, mentorship, self-care training, and communication skills training [9, 15].

In oncology one of the most frequently cited stressors is breaking bad news, particularly when oncologists feel inadequately trained in communication [17, 18]. Consequently, various approaches to communication skills training have been explored as a means to address this issue [19]. Organizational approaches involve programs centered on the work environment and often entail specific changes in work procedures or task restructuring. Their primary goal is to reduce job demand and enhance both job control and the level of participation in work-related matters. In summary, findings underscore the multifaceted nature of oncologist burnout [9, 15, 16], and a combined approach integrating both individual and organizational initiatives holds promise for achieving more substantial improvements in the well-being of oncologists.

### Knowledge gap and objectives

Given the limited understanding of the combined effects of individual and organizational approaches on doctors’ burnout levels, this study aimed to evaluate the impact of a novel approach: On-site Supportive Communication Training (On-site SCT) on burnout. The intervention has previously been described in details [20]. In brief, On-site SCT is a manual based three-day continuing educational intervention designed to enhance oncologists’ communication skills within a supportive learning environment. One key element of the intervention is that the doctors define their own learning goals. This aims to create an environment for professional development with a strong focus on each doctor’s individual work situation [21]. While the intervention primarily focuses on individual doctors, its interdisciplinary collaboration between in-house psychologists and oncologists emphasizes organizational integration. This collaboration is aimed to foster teamwork [21] and communication, while underscoring the organization’s commitment to addressing burnout comprehensively.

The study objectives were to (1) assess the effect of On-site SCT in enhancing communication skills among oncologists and thereby addressing burnout; (2) evaluate the feasibility of On-site SCT; and (3) assess doctors’ experiences with On-site SCT. The impact of the intervention on patient related outcomes as well as the doctors’ qualitative evaluation of the intervention will be reported separately.

## Methods

### Study design and setting

A randomized, controlled, multicenter study conducted at three Danish sites: Vejle Hospital, Aalborg University Hospital, and Zealand University Hospital. The project engaged seven experienced in-house psychologists to facilitate the intervention. As the protocol is already published [20], an overview focusing on the data relating to the doctors’ outcome measures is presented here. The study has been approved by the Research Ethics Committee at the University of Southern Denmark (23/19397).

### Participants

All medical doctors employed at the three participating oncology departments were screened for eligibility in 2023. Eligibility criteria included working in the outpatient clinic at least four days per month and providing informed consent. Doctors whose contracts expired during the study period or who could not participate fully, such as due to maternity leave, were excluded. Doctors were free to withdraw from the trial at any time without providing a reason. All dropouts were registered.

### Randomization

Doctors were randomly assigned to either the intervention or control groups in a 1:1 ratio using Research Electronic Data Capture (REDCap) version 13.1.25, employing a block size of 2/4. Stratification based on location was implemented to ensure an equal distribution of workload among psychologist and to allow explorative subgroup analysis based on site. The intervention duration varied between 3 and 4 months at each site, depending on the number of participating doctors.

### Intervention

**Fig. 1:**
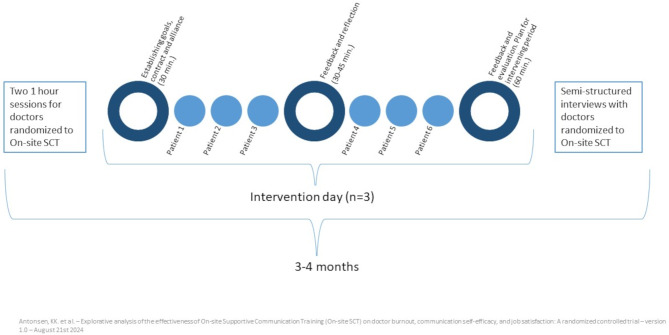
Overview of the intervention. On-site SCT: On-site Supportive Communication Training. Figure adapted from [20]

The psychologists observed doctor-patient consultations for one full working day at a time in the outpatient clinic. Three intervention days, each separated by approximately 3–4 weeks, were scheduled. The same psychologists were assigned to the same doctors throughout the intervention period. To ensure a common language between psychologists and doctors, a one-hour group session was held by the psychologist for all doctors randomized to the intervention before the first intervention day. Additionally, each doctor participated in a one-hour individual session with the psychologist to establish learning goal(s). An overview of the intervention is illustrated in Fig. 1 (originally published in our protocol paper) [20].

On intervention days, 30 min in the morning were scheduled for the psychologist and the doctor to prepare on the learning goal for the day. A 30 to 45-minute time slot was allocated for feedback after the completion of three to four consultations. At the end of the day, a 60-minute session was held for more thorough feedback and reflection. This joint reflection aimed to enhance doctors’ comprehension of their practices through double-loop learning and, if relevant, to devise and test novel approaches and strategies. During intervention days, the doctor’s schedule comprised six to seven patient consultations, with the psychologist observing.

### Assessments

The impact of On-site SCT on burnout was assessed using the Copenhagen Burnout Inventory (CBI) [22] part 2 on work-related burnout (seven questions including *“Do you feel worn out at the end of the working day?”*) and part 3 on patient-related burnout (six questions including *“Does it drain your energy to work with patients?”*), both scored on a scale from *always* or *to a very high degree* to *never/almost never* or *to a very low degree*. The CBI has shown excellent psychometric properties with Cronbach alphas for internal reliability of 0.85–0.87 [21, 22], and seems to be an appropriate measure of burnout in populations of health professionals [23]. All responses were converted to a numeric scale of 0-100, following CBI guidelines [22]. According to the CBI developers, differences of 5 points or more are considered significant for the individuals in question [22].

Communication self-efficacy (the individual’s own belief in his or her ability to perform a specified task successfully [24] was assessed using the questionnaire Self-efficacy in Health Communication (SE-12) [25]. Each question begins with the words: *“How certain are you that you are able to successfully…”* followed by a specific communication skill. The SE-12 Survey utilizes a numeric scale of 1–10, including the option “not relevant”. High levels reflect high self-efficacy. The SE-12 Survey is a reliable and partially validated instrument for assessment of clinicians’ self-efficacy in clinical communication before and after receiving communication skills training with a test-retest reliability of 0.71 (95% CI (0.66–0.76)) [25] and a person separation index (PSI) ranging from 0.92 to 0.94 [26].

Job satisfaction was assessed using five questions from the biennial employee satisfaction survey that is distributed among 25.000 Danish healthcare workers. The wording of the questions is outlined in Table 1. Job satisfaction is measured on a numeric scale of 1–7 (1 = “strongly disagree” and 7 = “strongly agree”).

**Table 1:** Effects of On-site SCT on burnout, job satisfaction, and communication self-efficacy

	Intervention group		Δ (95% CI)	Control group		Δ (95% CI)	*P*-value
	Baseline Mean (SD)	Evaluation Mean (SD)		Baseline Mean (SD)	Evaluation Mean (SD)		
Burnout							
CBI part 2: Work-related burnout	31.2 (16.5)	30.4 (17.3)	-0.9 (-4.3; 2.6)	38.1 (13.7)	34.8 (14.1)	-3.2 (-6.9;0.4)	0.34
CBI part 3: Patient-related burnout	22.0 (16.8)	23.6 (18.8)	1.6 (-2.1;5.3)	27.7 (15.6)	28.2 (15.9)	0.4 (-3.8;4.6)	0.67
Job satisfaction							
“I’m happy to go to work”	5.9 (1.0)	6.0 (0.9)	0.1 (-0.3;0.5)	5.8 (0.9)	5.7 (1.2)	-0.1 (-0.5;0.3)	0.39
“I experience good collaboration in my department”	5.8 (1.2)	5.9 (1.1)	0.1 (-0.3;0.5)	5.7 (1.1)	5.6 (1.1)	0.0 (-0.3;0.2)	0.52
“I have good opportunities for development (through new tasks, education, increased responsibility)”	5.4 (1.2)	5.7 (1.2)	0.4 (0.0;0.7)	5.5 (1.3)	5.5 (1.3)	0.0 (-0.4;0.3)	0.10
“Would you recommend others to apply for a job in your department?”	5.7 (1.4)	5.9 (1.2)	0.1 (-0.4;0.6)	5.8 (1.2)	5.7 (1.2)	0.1 (-0.5;0.2)	0.45
“Are you satisfied with your job overall, all things considered?”	5.6 (1.2)	5.7 (1.2)	0.1 (-0.2;0.5)	5.6 (0.8)	5.7 (1.1)	0.1 (-0.2;0.4)	0.84
SE-12							
Sum-score	94.84 (11.16)	100.77 (9.56)	5.92 (3.09;8.75)	91.56 (12.80)	97.29 (10.51)	5.73 (2.52;8.93)	0.93

*P*-values correspond to paired t-tests of change in the intervention group versus change in the control group. On-site SCT: On-site Supportive Communication Training. CBI: Copenhagen Burnout Inventory. SE-12: Self-efficacy questionnaire

The feasibility of the intervention was assessed by analyzing data from intervention days and comparing it to the planned schedule. Psychologists documented the time spent on shared morning preparation, as well as on feedback and reflection during lunch and in the afternoon. Additionally, they rated doctors’ motivation levels during intervention days on a scale from 1 to 10, with 10 being the highest.

Doctors’ reactions to On-site SCT were assessed using six questions inspired by Levels 1 (Reaction) and 2 (Learning) of The Kirkpatrick Model [27]. The Kirkpatrick Model is used to evaluate the efficacy of training within an organization and consists of four levels in total: Reaction, Learning, Behavior, and Results. “Reaction” measures whether the learners found the training relevant to their role, engaging, and useful. “Learning” measures whether the learner acquired the knowledge, skills, attitude, confidence, and commitment the intervention aimed to impart. “Behavior” is not included, as this level is measured by the SE-12, and “Results” is measured by the CBI. All questions were rated on a Likert-like scale from 1 (not at all) to 5 (very much).

Finally, the following data regarding the doctors were collected: Age, sex, primary language spoken at home, years since obtaining their university degree, job title, prior participation in communication training, and their feelings about being randomized to the intervention group.

### Statistical analysis

No power calculation was performed for the endpoints of this part of the study, as the number of eligible doctors was limited to those available in the departments. Feasibility was assessed comparing data from intervention days with the intended program for the intervention. Comparisons were based on descriptive statistics on doctors attendance in individual/group sessions, type and number of patient consultations, time spent on supervised reflection during the intervention days etc.

Hypotheses were tested using paired t-test in case of normally distributed data and Wilcoxon signed rank test in case of non-normality comparing the intervention and control groups. The level of significance was defined as 0.05. Histograms were used to investigate if data were normally distributed. All doctors that participated in two or more intervention days with a psychologist was defined as compliant to the intervention. Data were analyzed using an intention–to-treat approach. As a result of cross over (*n* = 1), per-protocol analyses was performed as sensitivity analyses. Explorative subgroup analyses were performed stratified by site and doctors’ experience (non-specialists vs. specialists).

## Results

### Eligibility and drop-out

**Fig. 2:**
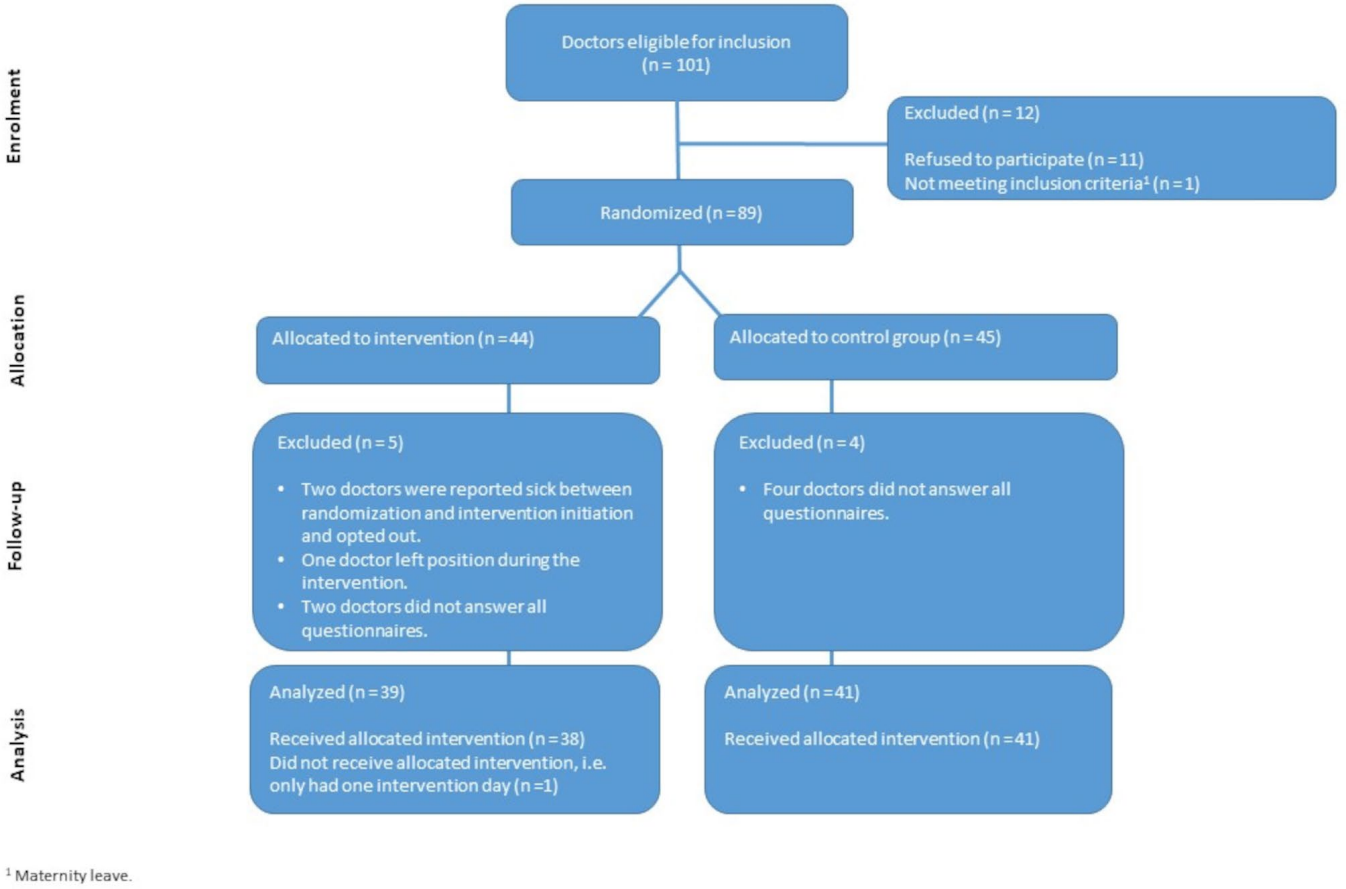
Participant flowchart

In the spring/summer of 2023 all doctors at the three participating sites were screened according to the in- and exclusion criteria. One hundred and one doctors fulfilled the criteria and were invited to participate in the study. Of the eligible doctors, 89 (89%) of the doctors provided informed consent. Forty-four doctors were randomized to intervention, and 45 doctors were randomized to the control group. Nine doctors were lost to follow up. Participation flowchart for the doctors is illustrated in Fig. 2.

### Participant characteristics

The baseline characteristics of the participating doctors (*n* = 80) are listed in Table 2. The mean age in the intervention group was 45 years (range 27–70 years), and the mean age in the control group was 47 years (range 30–75 years) There were fewer female doctors in the intervention group (*n* = 21; 54%) compared to the control group (*n* = 32; 72%). The mean number of years since graduation was 19.2 years (SD = 9.6) for the intervention group and 16.8 years (SD = 10.0) for the control group. The proportion of doctors who were specialists was higher in the intervention group (79.5%) compared to the control group (69.0%). Both groups had similar rates of previous non-mandatory communication training, with approximately 54% in each group. Feelings about being randomized to the intervention were obtained at baseline, and the median score was slightly more positive in the intervention group (median score: 7) compared to the control group (median score: 6).

**Table 2:** Baseline characteristics of the doctors in the intervention group, and in the control group

	Intervention group	Control group
Doctors, *n* (% of study population)	39 (48.8%)	41 (51.2%)
Age, median (min; max)	47.0 (30;75)	45.2 (27;70)
Sex, *n* (% female)	21 (53.9%)	31 (75.6%)
Seniority, mean years since graduation (SD)	19.2 (9.6)	17.0 (10.0)
Specialist, *n* (% yes)	31 (79.5%)	29 (70.7%)
Non-Danish language at home, *n* (%)	6 (16.2%)	9 (22.5%)
Previous non-mandatory communication training, *n* (%yes)	21 (53.9%)	23 (56.1%)
Feeling about intervention (median, IQR)*	7 (IQR 5–7)	6 (IQR 5–7)

*: Doctors’ feelings about being randomized to the intervention group on a scale from 1 to 7, where 1 was “I would feel really bad about it” and 7 was “I would feel really good about it”

### Effects on burn-out

For the evaluation of work-related burnout (CBI part 2), the intervention group had a mean score of 31.2 (SD = 16.5) pre-intervention, which slightly decreased to 30.4 (SD = 17.3) post-intervention, resulting in a change of -0.9 (95% CI: -4.3; 2.6). The control group had a mean score of 38.1 (SD = 13.7) pre-intervention which decreased to 34.8 (SD = 14.1) post-intervention, with a change of -3.2 (95% CI: -6.9; 0.4) (*p* = 0.34). Results are listed in Table 1.

For patient-related burnout (CBI part 3), the intervention group’s mean score was 22.0 (SD = 16.8) pre-intervention, which increased to 23.6 (SD = 18.8) post-intervention, resulting in a change of 1.6 (95% CI: -2.1; 5.3). The control group had a mean score of 27.7 (SD = 15.6) pre-intervention, which slightly increased to 28.2 (SD = 15.9) post-intervention, with a change of 0.4 (95% CI: -3.8; 4.6) (*p* = 0.67). Exploratory subgroup analyses were performed for all measures by site and doctors’ experience (specialists vs. non-specialists), with no changes in the listed results. One doctor was non-compliant (< 2 intervention days). Per-protocol analysis showed no changes in the results when including/excluding this respondent.

### Effects on job satisfaction

Across all five measures of job satisfaction, the analysis revealed no statistically significant differences between the intervention and control groups. Both groups showed minor improvements in their scores from baseline to evaluation, but no changes were sufficient to demonstrate a significant effect of On-site SCT on the doctors’ job satisfaction measures. However, the measure “I have good opportunities for development (through new tasks, education, increased responsibilities)” showed a trend toward improvement in the intervention group with an increase of 0.4 (95% CI: 0.0; 0.7) (*p* = 0.10), suggesting a potential positive effect of the intervention on perceived opportunities for development.

### Effects on communication self-efficacy

The mean difference in the change in SE-12 scores between the two groups was − 0.19 (95% CI: -4.42 to 4.03) (*p* = 0.93), indicating no significant difference in SE-12 score between the groups post-intervention. However, SE-12 scores increased in both groups from pre- to post-intervention. Although the difference between groups was not statistically significant, the increase from pre- to post-intervention might be clinically relevant, with the sum score increasing by nearly 6 points on average (5.92 and 5.73 in the intervention and control groups, respectively). The baseline sum score of SE-12 was 85.75 (SD = 11.64) for non-specialists and 95.63 (SD = 11.25) for specialists. Among non-specialists in the intervention group (*n* = 8), the baseline SE-12 score was 92.88 (SD = 11.41), compared to 81.00 (SD = 9.46) in the control group (*n* = 12).

### Feasibility of the intervention

85% (*n* = 33) of the doctors participated in the group session, while 92% (*n* = 36) participated in the individual session prior to the first intervention day. Almost all doctors were compliant with the intervention, with 38 out of 39 doctors having ≥ 2 intervention days. The psychologists reported a median motivation score of 9 out of 10 across all three days. The median number of patient consultations on intervention days was six, with very few additional acute patients (a total of 13 consultations, equating to 0.1 extra acute patient per intervention day per doctor). On average, there were 25 days between the first and second intervention day, and 28 days between the second and third.

**Fig. 3:**
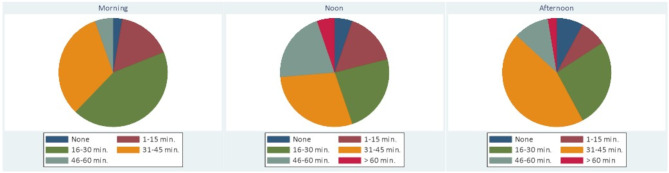
Time for reflection in the morning, at noon and in the afternoon on day 2

The amount of time spent on shared preparation in the morning, as well as on feedback and reflection during noon and in the afternoon is illustrated in Fig. 3. Day 1 and 3 had similar distributions.

The comparison between the planned schedule and the actual reported times reveals some variability in adherence. For the morning preparation, while the plan was for 30 min, the majority (43%) reported spending 16–30 min, with another significant portion (32%) spending 31–45 min, indicating a close alignment with the plan despite some sessions being shorter or longer. Midday feedback and reflection, planned for 30–45 min, saw 29% participants adhering to this timeframe, and a considerable number (24%) spending only 16–30 min, and 21% spending 46–60 min, showing a range of adherence with some exceeding the planned time. End-of-day feedback and reflection, planned for 60 min, had significant variability, the majority (45%) spent 31–45 min, and only 11% met the planned 46–60 min, with 26% spending 16–30 min and small portions either exceeding this time or having very short sessions.

### Doctors’ evaluation

**Fig. 4:**
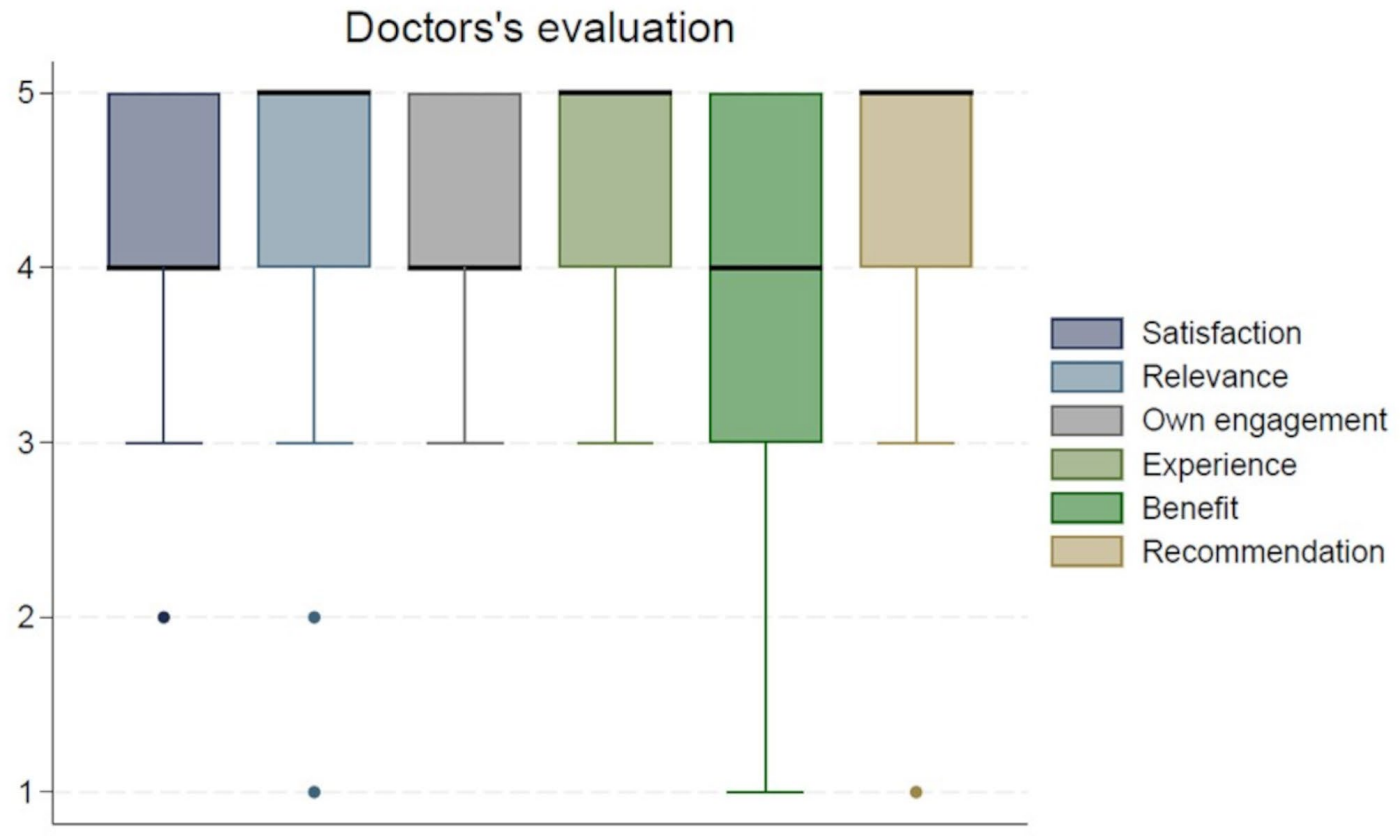
Doctors evaluation of the intervention on a 1–5 scale

All results are summarized in Fig. 4, which visually represent the distribution of responses across all six evaluation questions. These results indicate a positive reception overall. The median satisfaction score was 4, with 84% of the doctors rating their satisfaction as 4 or 5. In terms of relevance to daily practice, the median score was 5, with 85% of the doctors giving a relevance score of 4 or 5. The median engagement score during intervention days was 4, with 84% of doctors rating their engagement as 4 or 5. The experience of having the psychologist present in the consultation room was rated with a median score of 5, with 82% of doctors rating this aspect as 4 or 5 on the scale. The overall benefit of the intervention had a median score of 4, with 62% of doctors rating their benefit as 4 or 5. The likelihood of recommending the training to colleagues was rated with a median score of 5, with 84% of doctors giving scores of 4 or 5.

## Discussion

The objectives of this study were to assess the feasibility of On-site SCT, the effect of On-site SCT on work-related burnout, and doctors’ experiences with the intervention. On-site SCT showed a high level of feasibility in terms of planned sessions, although the time allocated for feedback and reflection varied considerably. We found no significant effect of On-site SCT on burnout, although doctors tended to report improved scores in communication self-efficacy (not statistically significant) and no effect on job satisfaction, aside from a non-significant improvement in perceived opportunities for development. The doctors reported an overall positive reception of the intervention.

Danish oncologists in this study reported a significantly lower prevalence of burnout compared to results from international literature [10, 12, 22, 28]. However, our findings align with another Danish study [11] involving 76 members of the Danish Society of Palliative Medicine. In that study the authors discussed several reasons for the low prevalence of burnout in their population, such as effective planning, supervision, and a shared understanding of the emotional demands within the field, which may also be applicable to our population. The low level of burnout at baseline raises the question of whether Danish oncologists might generally experience less job-related distress compared to their international counterparts, potentially introducing a floor effect for interventions like On-site SCT.

Various interventions for health care professionals aimed at enhancing communication have been shown to increase participants’ self-ratings on the SE-12 scale [29, 30]. A common limitation of these studies is the lack of a randomized, controlled design, which leaves an incomplete understanding of how SE-12 ratings evolve over time without specific interventions. In the SE-12 validation study [25], the authors tested the hypothesis that staff with previous communication skills training would rate themselves higher on the SE-12, which was confirmed with a delta of 4.28. Furthermore, they demonstrated a strong correlation between self-efficacy and field experience. This suggests that the change from pre-intervention to post-intervention seen in our intervention group could have a clinical impact, although this impact was not reflected in burnout scores. The slight imbalance in specialist status between groups at baseline suggests differing potentials for improvement, which may explain the increase in the control group. Additionally, the six-month gap between pre- and post-intervention evaluation, particularly for less experienced doctors, and the small sample size may have affected the study’s power to detect group differences.

The variation in actual time for feedback and reflection suggests some challenges in balancing clinical duties with participation in the intervention. However, the causes of these variations were not investigated in our study. Therefore, a limitation of our study is that we did not inquire about the doctors or psychologists’ perceived experience regarding the adequacy of time for feedback and reflection. As a result, we cannot draw conclusions on this aspect and its possible correlations to the lack of significant effect of the intervention. Participation in interventions like this may place additional stress on participating doctors, and we observed a non-significant increase in patient-related burnout scores post-intervention. To address this, we scheduled the post-intervention evaluation six months after randomization to allow time for reflection and adjustment; however, it may still have influenced the observed results.

## Conclusion

Our study found no significant changes in burnout among doctors participating in On-site SCT. Despite non-significant changes, the doctors reported an improvement in communication self-efficacy. The program showed high feasibility and received positive feedback from participating doctors.

### Perspectives

Although our training program included some organizational support elements, its primary focus remained on individual factors, such as the doctors’ personal learning goals, rather than on organizational aspects like collegial support, professional discussions, and work pressure. While these organizational factors could potentially influence whether a doctor experiences burnout, effective burnout prevention requires comprehensive strategies addressing departmental, organizational, and national dimensions. Our findings thus caution against assuming a causal relationship between short-term interventions, such as brief training programs and minor organizational changes, and a complex phenomenon like burnout, which influenced by numerous components. This caution aligns with insights from Shanafelt et al. [31], who emphasize the importance of executive leadership and comprehensive strategies in addressing doctor well-being.

In summary, while our intervention did not lead to measurable improvements in burnout, the high feasibility and acceptance indicate that such programs can be valuable. As a limitation, the study did not assess long-term outcomes beyond immediate satisfaction and likelihood of recommendation, limiting insights into sustained effects of the intervention. Therefore, On-site SCT will be implemented at the Department of Oncology, Vejle University Hospital, for a two-year trial period. This implementation aims to refine the process, ensure adequate time for feedback and reflection, and obtain long-term outcomes to gain insights into the possible sustained effects of the intervention.

## Data Availability

The datasets used and/or analyzed during the current study are available from the corresponding author on reasonable request.

## Abbreviations

CBI: Copenhagen Burnout Inventory
MBI: Maslach Burnout Inventory
On-site SCT: On-site Supportive Communication Training
REDCap: Research Electronic Data Capture
SE-12: Self-efficacy questionnaire (the number applies to the 12 items in the questionnaire)
